# Comprehending symmetry in epidemiology: A review of analytical methods and insights from models of COVID-19, Ebola, Dengue, and Monkeypox

**DOI:** 10.1097/MD.0000000000040063

**Published:** 2024-10-11

**Authors:** Siva Nanthini Shanmugam, Haewon Byeon

**Affiliations:** a Department of Digital Anti-Aging Healthcare (BK21), Inje University Medical Big Data Research Center, Inje University, Gimhae, Republic of Korea.

**Keywords:** epidemiological studies, fractional calculus, infectious diseases, mathematical models, public health initiatives

## Abstract

The challenge of developing comprehensive mathematical models for guiding public health initiatives in disease control is varied. Creating complex models is essential to understanding the mechanics of the spread of infectious diseases. We reviewed papers that synthesized various mathematical models and analytical methods applied in epidemiological studies with a focus on infectious diseases such as Severe Acute Respiratory Syndrome Coronavirus-2, Ebola, Dengue, and Monkeypox. We address past shortcomings, including difficulties in simulating population growth, treatment efficacy and data collection dependability. We recently came up with highly specific and cost-effective diagnostic techniques for early virus detection. This research includes stability analysis, geographical modeling, fractional calculus, new techniques, and validated solvers such as validating solver for parametric ordinary differential equation. The study examines the consequences of different models, equilibrium points, and stability through a thorough qualitative analysis, highlighting the reliability of fractional order derivatives in representing the dynamics of infectious diseases. Unlike standard integer-order approaches, fractional calculus captures the memory and hereditary aspects of disease processes, resulting in a more complex and realistic representation of disease dynamics. This study underlines the impact of public health measures and the critical importance of spatial modeling in detecting transmission zones and informing targeted interventions. The results highlight the need for ongoing financing for research, especially beyond the coronavirus, and address the difficulties in converting analytically complicated findings into practical public health recommendations. Overall, this review emphasizes that further research and innovation in these areas are crucial for addressing ongoing and future public health challenges.

## 1. Introduction

In recent years, the global health landscape has been significantly challenged by the emergence and resurgence of various infectious diseases. The COVID-19 pandemic, followed by outbreaks of Dengue, Ebola, and Monkeypox, has underscored the critical importance of epidemiological research in understanding and effectively managing these health crises. This study aims to delve into the analytical methodologies employed in epidemiology. It provides an in-depth examination of these methods, alongside offering insights derived from the epidemiological models of these 4 notable diseases. This comprehensive review seeks to enhance our understanding of the disease dynamics and the efficacy of various epidemiological strategies in addressing such global health concerns.

The recent global outbreaks of infectious diseases such as COVID-19 and Ebola have highlighted the necessity for effective and geographically targeted public health strategies. To optimize resource distribution and understand disease patterns, epidemiologists are increasingly employing spatial methods.^[1]^ However, the diverse application of these methods often leads to results that lack standardization, posing challenges for cross-national comparisons. This study aims to synthesize these advancements and insights, providing a comprehensive understanding of the analytical methods in epidemiology and their application in managing diseases like COVID-19, Ebola, Dengue, and Monkeypox. By integrating these findings, the research endeavors to offer an in-depth perspective on the utilization of epidemiological analytical techniques in the control and management of these significant health challenges.

Specifically, concerns were raised about the negligence of noncommunicable diseases, its reliance on specific databases like PubMed and CABI Digital Library while omitting others, and the necessity to standardize geographic nomenclature. This study has considerably reduced these gaps. This study has advanced the development of paper-based electrochemical biosensors for virus detection. It boosts the significance and dependability in predicting epidemic transmission.

## 2. Symmetry analysis

### 2.1. Symmetry analysis in epidemiological models

Symmetry analysis is a powerful tool for carefully creating numerical solutions for a given fractional differential problem.^[2]^ It is used to methodically produce numerical answers to differential equations involving fractions. This methodology facilitates a systematic investigation of the provided models, thereby augmenting the comprehension of disease processes. Numerical results and simulations were obtained using this novel technique of combining 2 operators. The technique is highly effective, dependable, and able to handle a wide range of scientific and technical problems. It helps to understand the way the virus spreads as well as to treat infections in a community when research is done in this area. With the developing COVID-19 pandemic, modeling research has increased dramatically in recent years. A disease’s trajectory, or whether it will become an epidemic, can be inferred from epidemiological models.^[3]^

### 2.2. Infectious agents

#### 2.2.1. COVID-19

The COVID-19 pandemic has exposed the prevalence of conservative and simplistic research in public health studies, news coverage, and policy formulation.^[4]^ Human respiratory infections caused by corona viruses can range in severity from mild to severe. Since its initial appearance in China in 2002, this Severe Acute Respiratory Syndrome Coronavirus, or SARS-CoV, has spread to 29 different nation worldwide.^[5]^ Humans contracted pneumonia, a lower respiratory illness that is deadly, from SARS-CoV. Since its discovery in Saudi Arabia in 2012, the Middle East Respiratory Syndrome Coronavirus, or Middle East Respiratory Syndrome Coronavirus, has spread to 27 countries in the area.^[6,7]^ The SARS-CoV outbreak claimed 774 lives worldwide, while the Middle East Respiratory Syndrome Coronavirus outbreak claimed 806 lives, according to the World Health Organization (WHO).^[8]^

#### 2.2.2. Ebola

The fatal Ebola virus disease is an illness that relatively rarely manifests itself. It was first discovered in Africa. People and nonhuman primates (including chimpanzees, gorillas, and monkeys) are both impacted by Ebola virus disease. In 1976, the Ebola virus was first discovered along the Ebola River in the Democratic Republic of the Congo. Since then, epidemics caused by the virus have periodically occurred in several African countries. Scientists are unsure about the exact source of the Ebola virus.^[9]^

#### 2.2.3. Monkeypox

The orthopoxvirus genus and Poxviridae virus family are home to the Monkeypox virus, which causes the respiratory disease known as Monkeypox. Human-to-human contact with infected humans or animals can spread the Monkeypox virus.^[3]^ An epidemic similar to smallpox occurred between 2 cynomolgus monkeys that were shipped from Singapore to Copenhagen in 1958. Over the next ten years, outbreaks were found in American captive monkey groups, although no human infections have been identified. During this time, the WHO’s members worked very hard to eradicate smallpox, and the majority of nations stopped producing the vaccine in 1980. Monkeypox instances in humans have been documented over time in numerous countries, even though the disease was first discovered in African nations.

#### 2.2.4. Dengue

Any 1 of the 4 dengue virus (DENV) variants can cause dengue, a viral disease spread by Aedes spp. Dengue Hemorrhagic Fever as well as Dengue Shock Syndrome, 2 potentially fatal consequences, can arise from an infection. However, a considerable percentage of cases are subclinical or inactive, generating insufficient distress for clinical presentation.^[10]^ The DENV has no particular therapy.^[11]^ Severe dengue cases necessitate hospitalization, but dengue patients require supportive treatment. Owing to the previously mentioned dengue-specific complications, the development of vaccines focuses on creating a tetravalent vaccination that is intended to offer long-term protection over all DENV serotypes.^[12]^

### 2.3. Recent developments

The fundamental reproduction number $R_{0}$ which represents the average number of further infections brought on by one infected person is estimated for the epidemic models that are created.^[13]^ $R_{0}$ is a crucial metric in epidemiology. This approximation aids in determining the likelihood of an epidemic breakout. This study makes use of the latest developments in electrochemical analysis by using square wave voltammetry and electron transfer redox prope $[FE[FE{\left(CN\right)}_{6}]{]}^{3-/4-}$ techniques. Pencil graphite electrodes modified with hydroxyapatite (HAP) and CuO demonstrate creative efforts to increase sensitivity and detection limitations.^[8]^

According to Mergenthale (2022), spatial epidemiology of transmissible illnesses demonstrates the wide range and constant development of research instruments and approaches. A vast range of spatial statistical techniques, from conventional choropleth mapping and models of linear regression to cutting-edge space–time cube models, are being used by researchers more and more. Notably, certain studies highlight cutting-edge visualization methods and stress the significance of accurate maps and visualizations, especially when it comes to space-time analysis.

A big step forward is the integration of Geographic Information System capabilities with global systems like District Health Information Software_2_. These systems offer a linkage to Geographic Information System systems, which makes basic spatial analysis easier as they are implemented globally. A wider spectrum of surveillance employees including public health researchers stand to gain from this integration, which will make it easier for them to carry out analytical work effectively. The change in direction towards this integration is recognition of the value of spatial analysis in improving the comprehension of illness patterns and assisting with well-informed decision-making.^[11]^

Also, Mergenthaler et al (2022) highlight the growing complexity of spatial statistical analysis by citing research that uses sophisticated space-time cube models and theoretical mathematical models.^[14]^ Although this intricacy indicates an increasing level of competence in the subject, the analysis points out a possible flaw, even the most complicated analyses frequently lack discourses on spatial patterns or clear visualizations. This highlights the necessity of striking a balance between the intricacy of the methodology and the efficient dissemination of geographic findings. Together, these advancements show that different spatial approaches and instruments are becoming more widely accepted. To improve the overall influence of spatial epidemiological studies on public health practices, they also highlight the significance of standardization, effective communication, and interaction with international systems.

### 2.4. Limitations and challenges

Population dynamics are complex and pose a problem for modeling infectious diseases like Ebola. Complexities brought about by variables including birth rates, migration, and demographic transitions might affect how accurate models are. One problem with treatment options is their efficacy. Comprehending the effects of medical interventions, such as vaccination and antiviral therapy, on the transmission of disease necessitates an in-depth comprehension of healthcare accessibility, infrastructure, and public compliance. It is frequently difficult to find accurate and thorough statistics on the prevalence of diseases, their rates of transmission, and other pertinent factors, especially in areas with poor access to healthcare.

Recent studies have acknowledged these shortcomings, which include the exclusion of research conducted outside of Health and Human Services potential underrepresentation of expenditures, and reliance on PubMed for research. The difficulty of maintaining financing for vaccine development is also covered. Ensuring successful antigen–antibody interactions and navigating the complexities of electrochemical measurements present additional challenges.^[8]^ The system has limits despite its advancements, such as the requirement for cautious parameter modifications and the possibility of electrode surface saturation at greater copper concentrations. One of the thirteen drawbacks is that the concentration is on 2 databases PubMed and CABI Digital Library while expert contacts and article bibliographies are left out. It is acknowledged that the search string is sensitive and excludes noncommunicable diseases. The use of spatial nomenclature varies, which highlights the necessity for standardization to improve the efficacy and intelligibility of spatial epidemiological research.

The accuracy and completeness of the data can impact how precise the models are. The strength of the public health infrastructure affects how well disease control methods work. The implementation and maintenance of interventions present difficulties in resource-constrained locations, which have an immediate impact on the accuracy of the modeling. Because of global connections, infectious diseases can spread fast across national borders. The models become more sophisticated when the interconnection of regions is modeled and the effect of global travel on the transmission of the disease is evaluated. Geographical location and climate have an impact on the spread of disease. A detailed grasp of these factors’ effects on the survivability of the virus and its dynamics of transmission is necessary to incorporate them into models.

The transmission of disease is significantly influenced by human behavior. Predictions become difficult when uncertainties are introduced into the modeling of individual behavior differences, adherence to preventative steps, and adherence to public health programs. It is frequently unclear when immunity develops and lasts after an infection or immunization. It is difficult to incorporate accurate immune profiles into models, yet doing so can have a big impact on how the disease is predicted to progress. Because it is impossible to precisely and consistently monitor infection rates, vector populations, as well as human recovery rates, there is uncertainty regarding to vector-borne diseases, such as dengue.

These qualities are typically incorporated into the modeling procedure as initial conditions or parameters. This data is required to construct more trustworthy models that enable us to comprehend the dynamics of this kind of illness and, consequently, provide suitable control recommendations. However, when simulating the spreading of infectious diseases in human populations, such experiments are frequently impractical, costly, or unethical, in contrast to many sciences where it is feasible to conduct multiple experiments to gather data and test hypotheses.^[11]^ Even with these complications, modeling is still an essential tool for comprehending and lessening the effects of infectious diseases like Dengue, Ebola, and others (Table 1).

**Table 1:** Overview of each diseases and distinctive challenges.

Disease	Context	Unique challenges in modeling and analysis
COVID -19	Global pandemic with rapid transmission^[8]^	High variability in transmission rates, asymptomatic cases, and varying outcomes
Ebola	Virulent hemorrhagic fever in Africa	Difficulty in controlling outbreaks due to remote locations and societal factors
Dengue	Mosquito-borne viral infection	Complex dynamics involving both human and mosquito populations
Monkeypox	Rare viral disease with zoonotic transmission	Limited understanding and sporadic outbreaks

## 3. Methods and models

### 3.1. Compartmental models

Brauer et al (2017) divide people into compartments in a compartmentalized disease transmission model as per single, discrete state variable. If the people within a compartment are contaminated,^[14]^ it is referred to as a disease compartment. It should be noted that this usage of the term “disease” goes beyond the clinical meaning and includes infection stages like exposed stages when infected individuals are not necessarily contagious.

Let $x\in R^{n}$ and $y\in R^{m}$ represent the subpopulations in each of the *n* disease compartments and *m* non-disease compartments. Furthermore, Brauer et al indicate the rate of disease progression, death, and recovery by $V_{i}$ and the rate of secondary infections increasing the *i*th disease compartment by $F_{i}$. Next, the compartmental model can be expressed in the following format (Brauer et al 2017).

$$\begin{array}{l} X^{\prime }=F_{i}\left(x,\;y\right)-V_{i}\left(x,\;y\right),\;\;i=1,\ldots \ldots \ldots .n,\;\;\; \end{array}$$ (1)

$$\begin{array}{l} Y^{\prime }=\;g_{j}\left(x,\;y\right),\;\;\;\;\;\;\;\;\;\;\;\;\;\;\;\;\;\;\;\;\;\;\;\;\;\;\;\;\;\;j=1,\ldots \ldots \ldots ..m.\;\;\; \end{array}$$ (2)

The classification of compartments into infected or uninfected and the breakdown of the dynamics as F and V might not be unique. Various decompositions align with distinct epidemiological analyses of the model.^[14]^

### 3.2. Stochastic models

The concept behind the stochastic branching process outline of a disease outbreak’s onset is the existence of an individual contact network, which can be represented as a graph with population members as vertices and contacts between individuals as edges. The 1950s and 1960s saw the development of Erdos and Renyi abstract theory, which laid the foundation for graph analysis. In recent times, it has gained significance in various domains including social interactions, computer networks, and the transmission of infectious illnesses. According to Brauer et al (2017), networks are bi-directional, meaning that disease can spread down an edge in either direction.^[14]^

An edge is a point of contact between vertices where an infection can spread. The degree of a vertex in a graph is the number of edges at that vertex. A graph’s degree of distribution is denoted as $\left\{p_{k}\right\}$, where $p_{k}$ represents the percentage of vertices with degree k. The generating function is $\;\;\;F_{0}\left(z\right)=\;\underset{k=0}{\overset{\infty }{\sum }}\;p_{k}Z^{k}$, where the power series converges for 0≤z≤1. A key concept in explaining how diseases spread is the degree distribution. It is possible to relax the assumption made by Brauer et al (2017) that every interaction between an infectious and a vulnerable person spreads infection.

### 3.3. Time model

According to Kiszewski et al (2020), National Institute of Health (NIH) funding is evaluated and innovations from the WHO’s COVID-19 vaccine scenario are identified. A bibliographic analysis method called the Technology Innovation Maturation Evaluation (TIME) framework is used in the study. This model evaluates the maturity of 7 key technologies since 1980 using an exponentiated logistic function. Kiszewski et al (2020) fitted time-series publication data to factors like growth rate and exponential growth midpoint. Publication trends are analyzed by the TIME model. The analysis includes NIH funding^[15]^ for pandemic illnesses such as dengue, corona virus, Zika, and Ebola. The emphasis goes beyond COVID-19 to illnesses that have the potential to become epidemics, emphasizing the larger background of vaccine research.

It stresses the value of technological maturity by drawing comparisons with past vaccine problems. The report explores vaccine development in the context of a 94% vaccine failure rate, highlighting the pressing need to produce a COVID-19 vaccine. Using a nonlinear least squares version of the Levenberg–Marquardt algorithm, the TIME model’s parameters were configured to time-series publication data. The equation takes the following form.

$$\begin{array}{l} log\;\;\;N=\frac{log\;\;\;L}{1+\;e^{-r(t-t_{0})\;\;\;}} \end{array}$$ (3)

where *t* is the time, *r* is the growth rate, N is the average number of publications, *L* represents the expected maximum number of publications, and *t*_0_ is the center of the exponential growth.

### 3.4. Age-structured epidemic models

Davydovych et al (2023) explore age-structured pandemic models by using age as the second time variable.^[13]^ These models show that the densities of vulnerable and infective individuals change with age in addition to geographical and time variations. These models’ governing equations, which depict the interactions between various age groups, are frequently integrodifferential. The governing equations are integrodifferential; therefore analytic conditions for the presence of traveling fronts in these models are investigated. Reasonable estimations in the form of partial differential equations were derived.

### 3.5. Cyclic voltammetry (CV) analysis

The pencil graphite electrode (PGE) has been successfully modified via HAP and copper oxide, as shown by the CV analysis. The modified voltammetric profiles demonstrate enhanced electron transport and a larger surface area, indicating modifications in the electrode’s behavior. The scanning electron microscope pictures, which show a rougher pattern on the electrode surface treated with HAP and copper oxide, provide more evidence for the modification process. Copper ions are confirmed to be present on the modified electrode surface by energy dispersive X-ray pictures, indicating that the interaction and modification using copper structures appear to be successful.

**Figure 1.**
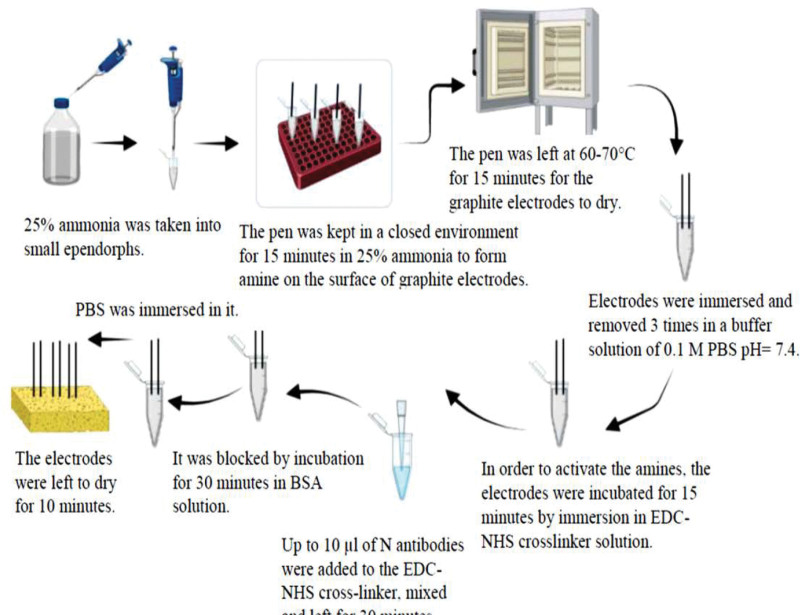
Functionalization of pencil graphite electrodes with COVID-19 nucleocapsid antibody^`^.^[12]^

Subsequent electrochemical investigations utilizing the generated electrodes entail immersing functionalized and modified pencil tips in varying concentration solutions to detect antigens. The analysis is facilitated by the electrochemical cell system, which consists of a reference electrode silver/silver chloride (Ag/AgCl) in aqueous potassium chloride (KCl), a working electrode (PGE), and a counter electrode (platinum wire). By testing the modified electrode’s efficiency using CV, characterizing the created electrodes, and investigating their electrochemical behavior. Improving analytical sensitivity for Severe Acute Respiratory Syndrome Coronavirus-2 (SARS-CoV-2) nucleocapsid protein detection is the aim. The steps required for the COVID-19 nucleocapsid antigen to bind to the surface of HAP and electrodes treated with copper oxide are depicted in Figure 1. Amine groups need to form on the PGEs’ surface for antibodies to attach to them.

With the use of an altered PGE, the biosensor platform that is being described offers a viable method for the quick and accurate identification of viral proteins, specifically the N protein of the SARS-CoV-2 virus.^[2]^ The utilization of electrochemical methods in conjunction with surface modification amplifies the biosensor’s sensitivity and selectivity, potentially rendering it useful for the identification of viral pathogens.^[8]^

### 3.6. Spatial heterogenity

The term “spatial heterogeneity consideration” describes how epidemiological models take into account regional variances or variations. The importance of taking into consideration spatial dynamics when simulating the spread of diseases, especially during the COVID-19 pandemic, is emphasized in the article’s setting. The COVID-19 pandemic showed notable regional differences in the virus’s distribution.

The concept of spatial heterogeneity acknowledges that disease transmission might differ depending on the place. The way an infection spreads over time and place within a population is better understood through equations. Models that take spatial heterogeneity into account show how epidemics spread in various geographic contexts more realistically. Several variables, including population density, commuting habits, and healthcare facilities, may contribute to the heterogeneity.^[1,13]^ A variety of geographic statistical techniques, such as choropleth maps, complicated space–time cube models, and linear regression models, are used in spatial epidemiology. Determining high-risk regions, gaining insights into illness trends, and supporting public health decision-making are the objectives.

### 3.7. Fractional calculus

One important methodology in the research is fractional calculus. In particular, Hasan (2023) discusses modeling non-integer order using the fractional differential Caputo operator, highlighting its relevance in examining the spread of Ebola virus outbreaks. With the use of this mathematical tool, one can portray dynamic systems with greater nuance by taking classical calculus to non-integer orders.^[16]^

### 3.8. Kermack–McKendrick model

The Kermack–McKendrick epidemic model was first formulated as follows and was published in Kermack and McKendrick (1927).

$$\begin{array}{l} v\left(t\right)=\;-X^{\prime }(t)\;\;\; \end{array}$$ (4)

$$\begin{array}{l} X^{\prime }\left(t\right)=\;-x\left(t\right)[\underset{0}{\overset{t}{\int }}\;A\left(s\right)v\left(t-s\right)ds+A\left(t\right)y_{0}]\;\;\; \end{array}$$ (5)

$$\begin{array}{l} Z^{\prime }\left(t\right)=\;\underset{0}{\overset{t}{\int }}\;C\left(s\right)v\left(t-s\right)ds+C\left(t\right)y_{0\;\;\;} \end{array}$$ (6)

$$\begin{array}{l} y\left(t\right)=\;\underset{0}{\overset{t}{\int }}\;B\left(s\right)v\left(t-s\right)ds+B\left(t\right)y_{0\;\;\;} \end{array}$$ (7)

In this case, the numbers, x(t) , y(t), and z(t) represent the susceptibles, infectious individuals, and recovered individuals, respectively. This also includes the recovery rate φ(s) for infection age *s* and the recovery rate Ψ(s) for the age s of infection.

$$\begin{array}{l} B\left(s\right)=\;e^{-\overset{t}{\underset{0}{\int }}\Psi \left(s\right)ds},\;A\left(s\right)=\;\varphi \left(s\right)B(s)\;\;\; \end{array}$$ (8)

It is considered that there won’t be any disease-related fatalities, keeping the population’s overall size steady. Despite not including the fundamental reproduction number in their study, Kermack and McKendrick were nonetheless able to arrive at a final size relation that is represented by the following Kermack–McKendrick model.

$$\begin{array}{l} log\frac{1-\frac{y_{0}}{N}}{1-p}=pN\overset{\infty }{\underset{0}{\int }}A\left(s\right)ds,\;\;\; \end{array}$$ (9)

Here, N is the total population size and *p* is the attack ratio


$$\begin{array}{l} p=1-\frac{X_{\infty }}{N} \end{array}$$

If, $S\left(t\right)=X\left(t\right),\;A\left(s\right)=B\left(s\right)=\;e^{-\gamma s},\;I\left(t\right)=\;\frac{N}{a}y\left(t\right).\;\;\;$

Therefore, the model can be reduced to system and a simple Kermack–McKendrick model as follows:

$$\begin{array}{l} S^{\prime }=\;-aS\frac{1}{N\;\;\;} \end{array}$$ (10)

$$\begin{array}{l} I^{\prime }=aS\frac{1}{N}-\gamma I.\;\;\; \end{array}$$ (11)

Davydovych et al (2023), acknowledge the historical importance of the work by Kermack and McKendrick, which established the groundwork for epidemiological mathematical modeling. This emphasizes how crucial basic concepts and historical context are to analytical methods. The transmission of infectious illnesses in a homogeneous community is described by this model.^[15]^ The mathematical model known as the Kermack–McKendrick work, or Kermack–McKendrick theory^[17]^ sheds light on the dynamics and spread of infectious illnesses within a population. The work is primarily linked to the 1927 publication “A Contribution to the Mathematical Theory of Epidemics” by W. O. Kermack and A. G. McKendrick. The population that the model assumes is homogenous, which means that every member of the population is thought to be equally susceptible to the disease.^[18]^ It is assumed that after just one infection, people become completely immune. A straightforward mathematical analysis is made possible by this simplification.

In comparison to an individual’s lifetime, the entire population is thought to have remained steady during the brief epidemic. Kermack and McKendrick first proposed the idea of an epidemic’s “threshold density.” There is no pandemic below this threshold. A minor rise in the infectivity rate above this point can trigger a major pandemic. The Kermack–McKendrick model indicates that epidemics may end before the susceptible population is completely depleted, in contrast to the widely held assumption that an epidemic ends when all susceptible individuals are infected. The link between infectivity, recovery, mortality rates, and population density affects termination.

### 3.9. Pontragin maximum principle

The Pontryagin maximum principle, named for the Russian mathematician Lev Pontryagin, is a cornerstone idea in the field of optimal control theory.^[3]^ When minimizing or maximizing a specific performance criterion is the goal, this approach is applied to determine the best control for a dynamical system. Consider a dynamical system described by a set of differential equations. The following is how the principle is expressed.^[19]^

$$\begin{array}{l} \dot{x}\left(t\right)=f(x\left(t\right),\;\;\;u\left(t\right),\;\;\;t)\;\;\; \end{array}$$ (12)

In this case, *u*(*t*) is the control input, *f* is a function characterizing the system dynamics, and *x*(*t*) reflects the status of the system at time *t*.

### 3.10. Optimal control analysis

Public health is unfortunately being severely impacted by the Monkeypox illness, particularly in less developed nations in West and Central Africa. It continues to have an impact in numerous fields. The importance of effective treatment and prevention strategies that can stop the disease from spreading has increased as a result. By modifying optimal treatment options, the symmetry model is defined in this context as an optimal control problem.

The influence of treatment and preventive approaches in halting the spread of Monkeypox is examined by Govindan (2023) using optimal control analysis.^[3]^ There are 2 control variables introduced, which stand for preventive (mask usage, social distancing, etc) and therapy measures. The aim is to minimize an objective function that accounts for medical and social distance expenses. The best control strategies are revealed by the application of Pontryagin maximal principle, which is utilized to find optimal solutions.

### 3.11. Taylor models

According to Lizarralde-Bejaranoa (2022), using the Taylor model technique is the only option to address the overestimation brought on by the wrapping effect and dependence problem. It combines symbolic computations with interval arithmetic.^[20]^ Taylor expansions and an enclosure are taken into account for the remaining to employ these techniques (Table 2).

**Table 2:** Brief overview of models and purposes.

Model	Description
Direct test	An approach for testing structural identifiability
Differential algebra	A method for assessing structural identifiability
Laplace transform	Utilized in testing if a model is structurally identifiable
Implicit function theorem	Approach for testing structural identifiability
Taylor series	Applied for structural identifiability testing
Profile likelihood	Methodology for testing structural identifiability
Output sensitivities	Technique for structural identifiability analysis

Formally, consider *a*(n + 1) times continuously partly differentiable function $f:\;D\subset R^{s}\to R.\;$


$$\begin{array}{l} T\;\;\;=\;\;\;\left(P,\;\;\;e\right)=\;\;\;P\;\;\;+\;\;\;e, \end{array}$$

It represents the Taylor model of a function *f* that is (n + 1) times constantly differentiable. *P* stands for the nth order Taylor polynomial of *f* on the expansion point $x_{0}\in D$, and *e* is a modest bounding set for the remaining portion of this approximation.

$$\begin{array}{l} f\left(x\right)-P\left(x-x_{0}\right)\in e,\;\;\forall x\in D\;\;\;where\;\;\;x_{0\;\;\;}\in D\;\;\; \end{array}$$   (13)

### 3.12. Structural identifiability analysis

A structural identifiability analysis of a model can be used to assess whether it is possible to retrieve the optimal model parameters distinctively given the assumption that the data is noise-free.^[11]^ This analysis is not dependent on the accuracy of the experimental data; rather, it depends entirely on the model structure. Using the Identifiability Analysis module in Mathematica, a structural identifiability analysis is performed on the model to ascertain whether beginning conditions and parameters can be specifically identified from the weekly observed dengue cases.

The results by Lizarralde-Bejarano (2023) show that without more information, some characteristics and initial conditions like the mosquito recruitment rate and initial conditions for susceptible, disclosed, and infected mosquitoes cannot be identified locally. This emphasizes how additional data must be collected to increase the model’s identifiability. Based on the given data, the structural identifiability analysis identified some parameters and beginning conditions that might not be locally recognizable. This highlights how crucial it is to gather more data to improve identifiability, particularly for variables about mosquito populations.

### 3.13. Local sensitivity analysis

The basic reproduction number $\left(R_{0}\right)$ is the focus of the local sensitivity analysis, which investigates the effects of changing various factors on the incidence of secondary dengue cases.^[11]^ The most significant parameters are those that relate to rates of transmission $(\beta_{m},\;\beta_{h}),\;$ human recovery rates $\left(\gamma_{h}\right)$, and mosquito mortality rates $\left(\mu_{m}\right)$. For the localities under examination, the study offers insightful information about the relative significance of these criteria in forecasting the changing patterns of dengue spread.

### 3.14. Validating solver for parametric ordinary differential equations

A computer method for solving ordinary differential equations (ODEs) that offers assurances of the accuracy of the results is called the Verified ODE Solver, or validating solver for parametric ODE (VSPODE). The unique aspect of VSPODE is how it addresses uncertainties related to parameters and initial circumstances in mathematical models by applying interval arithmetic. Interval arithmetic is used by VSPODE to handle uncertainty. The tool can generate solution intervals that give constraints on feasible trajectories by defining intervals as opposed to utilizing the value of points for parameters and initial circumstances. The tool solves problems like the wrapping effect, the curse of dimensionality, and the dependence problem.^[11]^ The goal of VSPODE is to manage overestimation at every stage of integration. This is essential to developing solutions that strike a balance between computing economy and precision without being unduly conservative.

### 3.15. Power-law Kernels

Using a power law kernel, Hasan et al (2023) highlight the two-step polynomial Newton technique in particular. The selection of this kernel is essential for describing the behavior of the infectious disease and its modes of transmission. According to Hasan, this model which uses a power law kernel is reliable and able to replicate theoretical circumstances.

### 3.16. Comparison with Caputo derivative operator

Hasan (2023) presents a comparative analysis using the Caputo derivative operator. This compares and contrasts the outputs of the conventional Caputo derivative operator with the fractal fractional Caputo operator. The dynamic behavior of the disease can be better understood by doing a comparative study like this on.^[3,16]^ Farman (2023) using the modeling non-integer order in the mathematical representation of Ebola virus infections with the fractal fractional Caputo operator. By including fractional orders in standard calculus, this operator enables a more precise and intricate depiction of the dynamics of the disease.

### 3.17. Atangana–Baleanu fractional derivative (model for Monkeypox)

Gunasekar et al (2023) employ the Atangana–Baleanu fractional derivative to alter the conventional epidemiological model for Monkeypox. For both people and rats, the differential equations describing the dynamics of susceptible, infected, treated, and recovered populations are solved using the fractional derivative.^[3]^

The left Atangana–Baleanu fractional derivative denoted as $D_{AB}^{\rho }f\left(t\right),$ is defined as the RL (Reimann–Liouville) integral. For a function f(t), the fractional derivative is given by the integral formula involving the gamma function and a parameter ρ.

$$\begin{array}{l} \left(D_{AB}^{\rho }f\right)\;\left(t\right)=\frac{1}{\Gamma \left(\rho \right)}\overset{t}{\underset{0}{\int }}{\left(t-s\right)}^{\rho -1}f\left(s\right)ds\;\;\; \end{array}$$ (14)

The incorporation of the Atangana–Baleanu fractional derivative into the epidemiological modeling of Monkeypox brings a novel and distinctive element. To represent the fractional aspect of the temporal evolution in the transmission of Monkeypox, this adds a fractional order, represented by ρ. The purpose of this method is to better represent the subtleties and complexities of the dynamics of disease transmission. The temporal evolution of the infectious disease can be described in greater detail thanks to the fractional calculus. The stability analysis of these points is affected by the derivative’s fractional character, which helps to provide a more thorough insight into the disease’s long-term behavior.

### 3.18. Novel model framework for Monkeypox transmission

**Figure 2.**
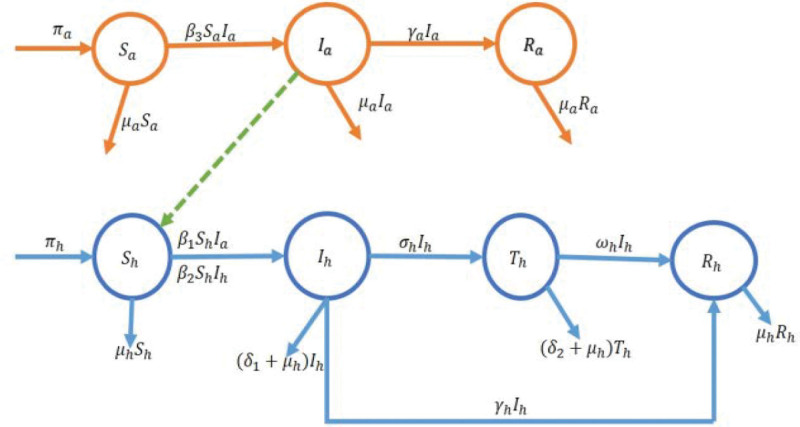
Flowchart of Monkeypox transmission between humans and rodents.^[2]^

According to the novel model as shown in Figure 2, rodents and humans can contract Monkeypox. In contrast to other Monkeypox models, this one takes into account both humans who have received treatment and rodents who have recovered naturally. The current model is new since it takes into account every scenario in which 2 species could interact. $N_{h}(ℵ)$, susceptible humans $S_{h}(ℵ)$, infected humans $I_{h}(ℵ)$, treated humans $T_{h}(ℵ)$, and recovered humans $R_{h}(ℵ)$ represent the entire human population at time *t*. $N_{h}(ℵ)=S_{h}(ℵ)+I_{h}(ℵ)+$$T_{h}(ℵ)+R_{h}(ℵ)$. Overall, there are rodents in the population $N_{a}(ℵ)$, susceptible rodents $S_{a}(ℵ)$, infected rodents $I_{a}(ℵ)$, and recovered rodents $R_{a}(ℵ)$. The formula for $N_{a}(ℵ)$ is

$$\begin{array}{l} S_{a}(ℵ)+I_{a}(ℵ)+R_{a}(ℵ).\;\;\; \end{array}$$ (15)

### 3.19. Stability analysis

A stability study of the equilibrium points, examining the local asymptotic stability of the Endemic and Monkeypox-free equilibrium points, is made possible by the fractional model.^[3]^ Understanding the disease’s long-term behavior and the effects of control strategies depends on the stability analysis. A stability study was conducted by Gunasekar and Manikandan (2023) for a model of Monkeypox transmission across populations of non-human primates and humans.

### 3.20. Interval analysis

Ramon Moore was primarily responsible for the development of interval arithmetic in the 1960s as he did so to rigorously take into consideration rounding errors associated with mathematical calculations.^[11]^ The set of closed intervals in ℝ serves as the foundation for this theory.

$$\begin{array}{l} I=\;\{\;X\;\;\;=\left[\underline{x,\;\;\;}\bar{x}\right]\;|\;x\leq \bar{x}\wedge \underset{\text{-}}{x},\;\;\;\bar{x}\in \text{ℝ}\}\;\;\; \end{array}$$ (16)

Interval analysis has 2 key drawbacks such as the wrapping effect and the dependency problem. When a variable appears more than once in the synthesis for a function, it is called a dependency problem. When the result cannot be an interval or box in intermediate computation phases and must be enclosed in an interval or box, the wrapping effect occurs.

### 3.21. Dengue fever epidemiology

The development of epidemic models^[21]^ of disease transmission aims to comprehend the patterns of pathogen–host interactions. Most of the proposed models attempt to include factors that focus on various aspects of the disease (such as co-infection, bite rate, and transmission capacity) as well as some biological aspects of the mosquito vector. These perspectives range from microscopic within-host dynamics to macroscopic transmission dynamics at population level. After being substantiated by data, these frameworks are expanded to address inquiries regarding the consequences of the accessible control methods, rendering them efficacious instruments for public health decision-making.^[16]^

### 3.22. Fractional order model of Ebola with treatment

A deterministic model is provided by Hasan et al to help understand the dynamics of the Ebola virus transmission. Hasan and Yadav (2023) using models to study the origins and recurrence of epidemics.^[9]^ This is a brief overview of some of the main features of the compartmental mathematical epidemic model that Hasan (2023) developed to describe the spread of viruses.^[22]^ A system of nonlinear ODEs describes the epidemic Ebola virus treatment model^[4]^ as follows.


$$\begin{array}{l} CD_{0,t}^{v1,v2}S\left(t\right)=\Lambda -\left(\beta_{I}I+\;\beta_{H}H+\beta_{D}D\right)S-\left(\tau +\mu \right)S, \end{array}$$

$$\begin{array}{l} CD_{0,t}^{v1,v2}E\left(t\right)=S\left(\beta_{I}I+\;\beta_{H}H+\beta_{D}D\right)S-\left(\mu +\tau +\delta \right)E \end{array}$$

$$\begin{array}{l} CD_{0,t}^{v1,v2}I\left(t\right)=\delta E-\left(\mu +\gamma \right)I, \end{array}$$

$$\begin{array}{l} CD_{0,t}^{v1,v2}H\left(t\right)=\gamma I-\left(\mu +\lambda +\alpha \right)H, \end{array}$$

$$\begin{array}{l} CD_{0,t}^{v1,v2}R\left(t\right)=\alpha H-\left(\mu +\tau \right)\theta D, \end{array}$$

$$\begin{array}{l} CD_{0,t}^{v1,v2}D\left(t\right)=\lambda H-\theta D.\;\;\; \end{array}$$ (17)

Where *S*(0) = *S*0 ≥ 0, *H*(0) = *H*0 ≥ 0, *E*(0) = *E*0 ≥ 0, *I*(0) = *I*0 ≥ 0, *R*(0) = *R*0 ≥ 0, *D*(0) = *D*0 ≥ 0 are the initial conditions.

### 3.23. Hamiltonian structure/systems

Being Hamiltonian is generally considered to be a highly restricted feature for a dynamical system. But Ballesteros et al (2020) have just demonstrated that the generalized susceptible-infectious-recovered (SIR) model is completely Hamiltonian.^[23]^ This implies that a more thorough comprehension of this outcome ought to be achievable. As it happens, this is only an example of a much broader result that says that any compartmental model that has a constant population is a Hamiltonian system.^[11]^

### 3.24. SIR model

For the entire population, which consists of humans and rodents, Manikandan (2023) proposed and dynamically analyzed the SIR model as it relates to pox-like infection.^[24]^ In epidemiology, the SIR framework^[25]^ is a compartmental model that is frequently used to comprehend how infectious illnesses propagate throughout a community.^[12,26]^ The ODEs that characterize the movement of people between these divisions over time serve as the foundation for the model’s operation. Usually, characteristics like the transmission rate and recovery rate are involved in these transitions.^[27]^

**Figure 3.**
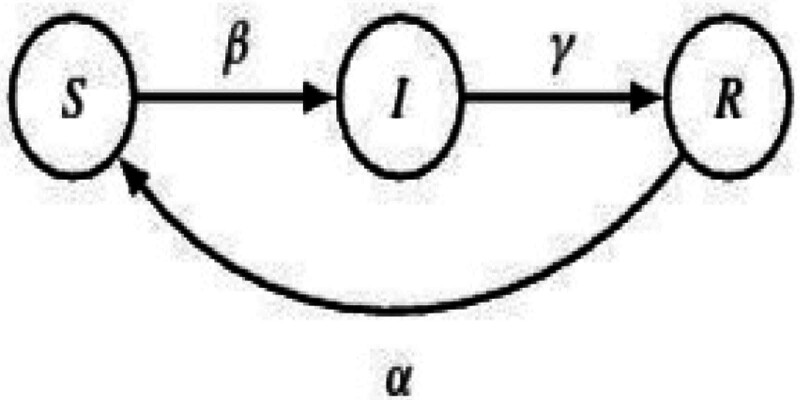
State flow diagram of simple epidemiological SIR type model.^[4]^

Susceptible S, Infected I, and Recovered R are the stages associated with the disease. The rates of infection (β), recovery (γ), and declining immunity (α) characterize the changes in a disease-related state for a host population of N individuals. The SIR model^[28]^ sheds light on how the COVID-19 population flows across these compartments at different points in time as shown in Figure 3. It offers information on infection patterns, possible case peaks, and the general effects on public health.

Kermack and McKendrick extended the basic SIR system to include more general interactions among the susceptible, infected and recovered compartments.^[23]^

$$\begin{array}{l} \dot{S}=\;\;\;-\beta SI-\varphi_{S\to I}\left(S,\;\;\;I,\;\;\;R\right)-\varphi_{S\to R}\left(S,\;\;\;I,\;\;\;R\right),\;\;\; \end{array}$$  (18)

$$\begin{array}{l} \dot{I}=\;\;\;\beta SI-\alpha I+\;\;\;\varphi_{S\to I}\left(S,\;\;\;I,\;\;\;R\right)-\varphi_{I\to R}\left(S,\;\;\;I,\;\;\;R\right),\;\;\; \end{array}$$ (19)

$$\begin{array}{l} \dot{R}=\;\;\;\alpha I+\varphi_{S\to R}\left(S,\;\;\;I,\;\;\;R\right)+\;\;\;\varphi_{I\to R}\left(S,\;\;\;I,\;\;\;R\right),\;\;\; \end{array}$$  (20)

where the transfer function via compartment A to compartment B, which can generally be any function of S, I and R, is represented by the symbol $\varphi_{A\to B}\left(S,\;I,\;R\right).$

### 3.25. Endemic SIR model

An endemic disease is distinguished by a nonpermanent immunization, meaning that infected persons can reinfect after a long enough period.^[23]^

From the basic SIR system is given by Hamiltonian system, H(S, I, R)=S+I+R and the poisson algebra defined with fundamental poisson brackets


$$\begin{array}{l} \left\{I,\;R\right\}=-\alpha I+\beta SI+\varphi_{2}\left(S,\;I\right), \end{array}$$

$$\begin{array}{l} \left\{S,\;R\right\}=-\beta SI+\;\varphi_{1\;}\left(S,\;I\right), \end{array}$$

$$\begin{array}{l} \left\{S,\;I\right\}=0. \end{array}$$

Where $\varphi_{1\;}=-\varphi_{S\to I}-\varphi_{S\to R}$ and $\varphi_{2\;}=\varphi_{S\to I}-\varphi_{I\to R}.\;$

Therefore, Hamilton equations becomes,


$$\begin{array}{l} \dot{S}=\left\{S,H\right\}=\;-\beta SI+\varphi_{1\;}\left(S,\;I\right), \end{array}$$

$$\begin{array}{l} \dot{I}=\left\{I,H\right\}=\;\beta SI-\alpha I+\varphi_{2}\left(S,\;I\right), \end{array}$$

$$\begin{array}{l} \dot{R}=\left\{R,H\right\}=\;\alpha I-\varphi_{1\;}\left(S,\;I\right)-\varphi_{2}\left(S,\;I\right). \end{array}$$

To recover a basic model that captures this feature, set $\varphi_{1\;}\left(S,\;I\right)=\;\mu I$ and $\varphi_{2}\left(S,\;I\right)=\;-\mu I$.

The ODE system turns into

$$\begin{array}{l} \dot{S}=\;-\beta SI+\;\mu I,\;\;\; \end{array}$$ (21)

$$\begin{array}{l} \dot{I}=\;\beta SI-\left(\alpha +\;\mu I\right),\;\;\; \end{array}$$ (22)

$$\begin{array}{l} \dot{R}=\;\alpha I.\;\;\; \end{array}$$ (23)

### 3.26. Susceptible-infectious-recovered-deceased (SIRD) model

The SIRD model is the most well-known generalization of the SIR model that assumes that the diseased subpopulation has a nonzero mortality rate.^[29]^ In contrast to the SIR model, which assumes that the recovered group gains immunity from the disease, the SIRD model considers the following assumptions: there is no cure or immunity, and some infected people of the subpopulation will die at a specific rate. The recovered subpopulation will also not remain immune and may become infected again.^[13]^

### 3.27. Susceptible-exposed-infected-recovered (SEIR) model

A popular compartmental model in epidemiology is the SEIR model^[4,26]^ (Table 3). Four divisions are created by it: Recovered (R), Infectious (I), Exposed (E), and Susceptible (S). People go through these stages as they become infected and heal from the illness.^[3]^ One single-strain model used to comprehend the dynamics of dengue transmission is the SEIR model.^[24]^ The degree to which the SEIR model fits the observed data is evaluated using statistical measures like the Root Mean Square Error and Deviance Information Criterion.

**Table 3:** Comparison of different models using statistical indicators.

Model	Model (DIC)	Model (RMSE)	Findings
SEIR	Yes	Moderate	Overestimates 2002 outbreak, underestimates peaks
SEIAR	No	Similar to SEIR	Similar to the dynamics of SEIR, small overestimation
SEIR2	No	Similar to SEIR	Does not improve over single-strain models.
SEIR2psi	Yes	Lowest RMSE	Reproduces 2007 epidemic well, lower over dispersion

DIC = deviance information criterion, RMSE = root mean square error, SEIAR = susceptible-exposed-infected-asymptomatically-infected-removed model, SEIR = susceptible-exposed-infected-recovered model, SEIR2 = two-strain SEIR model, SEIR2psi = advanced two-strain SEIR model.

### 3.28. Reaction-diffusion equations

The reaction-diffusion equations can be employed to simulate the diffusion-based spread of the diseased population. The dynamics of spatially extended systems where both reaction and diffusion processes are involved in component interactions are described as reaction-diffusion systems. These models have been extensively used to study the behavior and spread of populations or substances in a variety of domains, including biology, chemistry, physics, and epidemiology. Reaction-diffusion systems are a useful tool for studying the spatial spread of infectious diseases when considering epidemic modeling.

$$\begin{array}{l} \frac{\partial s}{\partial t}=d_{s}\frac{\partial_{s}^{2}}{\partial x^{2}}+\mu_{nd}-\alpha_{i}\frac{si}{1+qi^{2}}-\mu_{nd}S,\;\;\; \end{array}$$ (24)

$$\begin{array}{l} \frac{\partial e}{\partial t}=d_{e}\frac{\partial_{e}^{2}}{\partial x^{2}}+-\alpha_{i}\frac{si}{1+qi^{2}}-\;\left(\gamma +\mu_{e}+\varepsilon +\mu_{nd}\right)e,\;\;\; \end{array}$$ (25)

$$\begin{array}{l} \frac{\partial i}{\partial t}=d_{i}\frac{\partial_{i}^{2}}{\partial x^{2}}+\gamma e-\left(\sigma +\mu_{i}+\mu_{nd}\right)i,\;\;\; \end{array}$$ (26)

$$\begin{array}{l} \frac{\partial r}{\partial t}=d_{r}\frac{\partial_{r}^{2}}{\partial x^{2}}+\sigma i+\epsilon e+\mu_{nd}r\;\;\; \end{array}$$ (27)

An investigation is conducted into COVID-19 dissemination using a reaction-diffusion model. By including the effects of individuals from different compartments moving randomly, the model is a spatial expansion of the SEIR model with nonlinear incidence rates (subpopulations). One extremely specific example of a model is the diffusive SEIR model. In fact, rather than using the general function m_2_ (i), Hasan, (2023) employs the particular function $\frac{1}{1+qi^{2}}$. It should be emphasized, though, that the system now includes a new term, ee which accounts for exposed people’s COVID-19 immunity.

### 3.29. Paper-based electrochemical biosensors for virus detection

Paper is usually used as the supporting material for the biosensor, together with an electrode for signal transduction and a bio-recognition element for interactions specific to viruses. The immobilization of a bio-recognition component such as antibodies or aptamers onto the paper substrate is the first step toward functionality. This component specifically captures the intended virus by acting like a molecular recognition tool. When a measurable signal is produced by the binding event, usually through variations in current or voltage, electrochemical transduction enters the picture.

**Figure 4.**
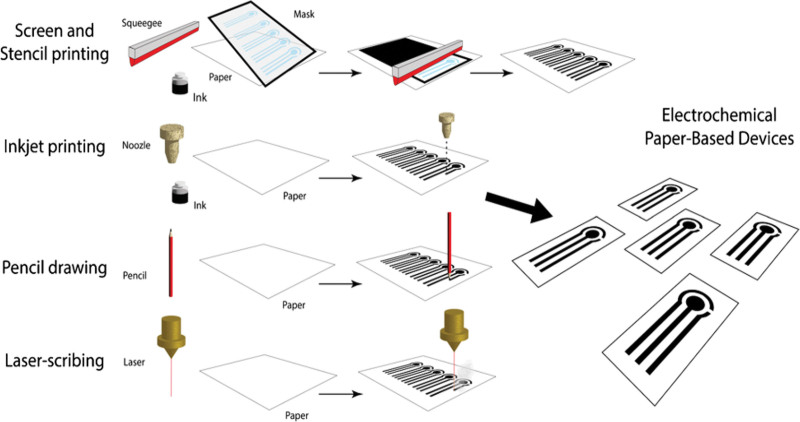
Representation of electro-chemical paper-based device fabrication.^[30]^

Firstly, these biosensors incorporate the selectivity of electrochemical approaches with the adaptability of paper substrates (Fig. 4). To create one platform for targeted viral detection, the development entails making electrodes on paper with conductive materials. By integrating bioreceptors onto the paper’s surface, this method frequently enables virus biomarker binding that is selective. Integrating nanomaterials such as nanoparticles to boost signal strength and enhance sensor performance is one important component. The produced electrochemical signal is then measured, allowing the concentration of the virus to be ascertained. Paper and nanomaterials work together to improve the sensitivity of the biosensor, making it possible to detect viruses at less concentrated levels. The porous characteristic of paper increases the surface area available for biofunctionalization, hence improving the sensitivity of the sensor.

### 3.30. Multiplexed paper sensor for dengue serotype detection

**Figure 5.**
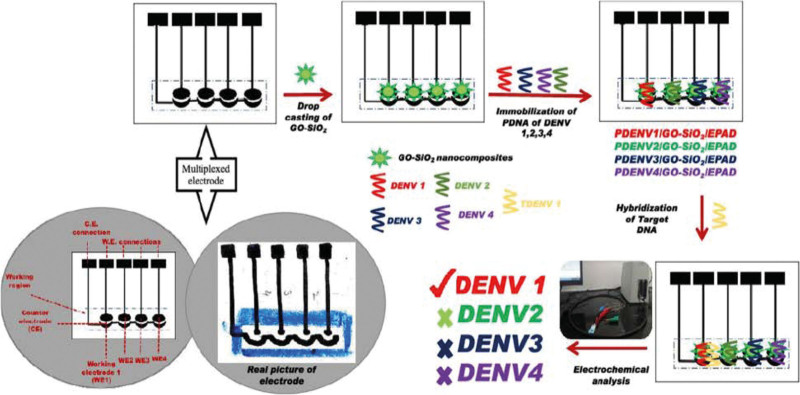
Multiplexed paper sensor for dengue serotype detection.^[30]^

To fabricate multiplexed paper sensors for dengue serotype detection, a diagnostic instrument that can distinguish between many DENV serotypes on a single platform must be developed (Fig. 5). This strategy makes testing more thorough and targeted, which is essential in areas where different dengue serotypes coexist. The sensor becomes affordable, readily deployable, and accessible by utilizing paper-based technology, which makes it an invaluable tool for accurate dengue diagnosis.

### 3.31. Electrochemical biosensor for rapid detection of SARS-CoV-2

**Figure 6.**
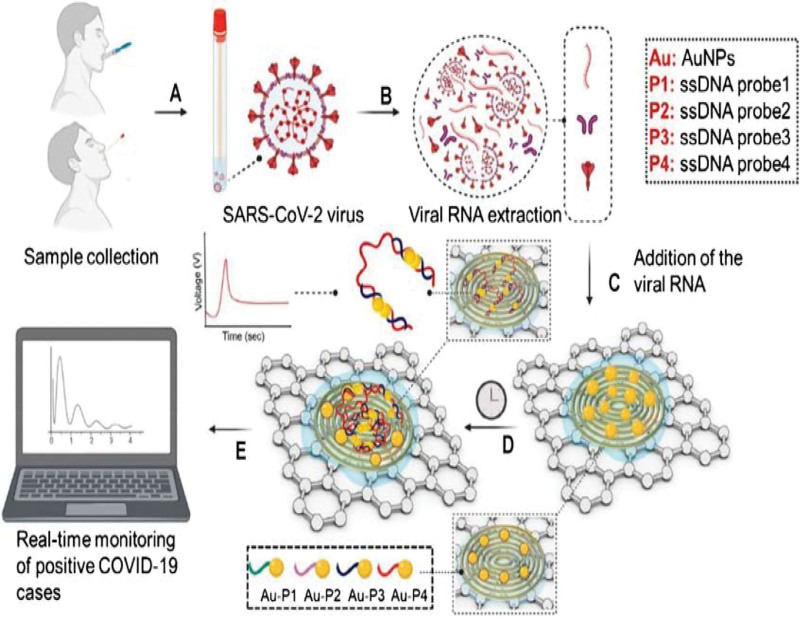
Electro-chemical biosensor for rapid detection of SARS Cov-2.^[30]^ SARS-CoV-2 = Severe Acute Respiratory Syndrome Coronavirus-2.

An electrochemical biosensor that uses antisense ologonucleotides for SARS-CoV-2 detection provides quick and accurate virus identification, as shown in Figure 6. Nucleotide sequences known as antisense oligonucleotides supplement the viral ribonucleic acid and enable targeted detection. Previous detection methods lacked the speed and specificity needed for effective early intervention. This electrochemical biosensor addresses these shortcomings resulting in rapid, specific and cost-effective diagnostic tools. The electrochemical component improves sensitivity and accuracy, yielding outcomes that are dependable and timely (Table 4). This method is essential, particularly in situations where prompt detection of SARS-CoV-2 is necessary for prompt intervention and management.

**Table 4:** Functionality of multiplexed paper sensor and electrochemical biosensor.

Multiplexed paper sensor for dengue serotype detection		Electrochemical biosensor for rapid detection of SARS-CoV-2	
Sample application	A patient’s blood serum is applied to the paper sensor	Target RNA binding	Antisense oligonucleotides on the sensor are designed to bind specifically to the RNA sequences of SARS-CoV-2.
Serotype-specific recognition	Sensor contains specific elements that interact with proteins or genetic markers unique to each dengue serotype	Electrochemical signal generation	Binding induces an electrochemical change (current or voltage shift) indicating the presence of the virus
Colorimetric or electrochemical signal	Interaction triggers a color change or generates an electrochemical signal Visible to the naked eye or measurable through a simple device.	Rapid detection	The Electrochemical nature enables quick and precise identification Offering results in a shorter timeframe compared to traditional methods.
Multiplexed output	Sensor displays distinct responses as per the presence of different dengue serotypes on a single platform	Point-of-care applicability	Designed for use at the point of care Biosensor provides a user-friendly, on-site diagnostic tool for rapid SARS-CoV-2 detection.

RNA = ribonucleic acid, SARS-CoV-2 = severe acute respiratory syndrome coronavirus-2.

### 3.32. SEIAR model (susceptible-exposed-infected-asymptomatically infected-removed)

The SEIAR model, which is an expansion of the SEIR model, has a separate compartment for people who are asymptomatic (A) enabling better simulation of infection dynamics. This makes it possible to mimic infected people who don’t exhibit any symptoms. To facilitate model comparisons, the SEIAR model takes into account the total amount of symptomatic cases for DENFREE data.

The SEIAR model often lacked the complexity to accurately represent asymptotic cases and multiple pathogen strains.^[31]^ An expansion of the SEIR model, the two-strain SEIR model presents the idea of 2 interacting strains of an infectious agent. It is especially important when several pathogen strains influence how the disease progresses. To measure the interaction between the 2 strains, the advanced two-strain SEIR model including interaction parameter ψ adds a third parameter, ψ. The goal of this model is to represent more intricate details of the dynamics of transmission incorporating several strains.^[10]^

### 3.33. Forward-backward sweep method

Numerical approaches, such as the forward backward sweep method, are critical for improving model parameters to match real-world data. This is to forecast how the disease would behave over a specific time frame involves resolving the differential equations of the model.^[3]^ The simulated outcomes generated by the forward sweep are compared with actual data that has been seen in the reverse sweep. It minimizes the discrepancies between the model predictions and the actual data, enhancing the model’s accuracy. Forward and backward sweeps are performed iteratively till a set of parameters is determined that best fits the data that was collected. It often results in less accurate predictions. By improving the model’s parameters, this optimization technique helps to improve the reliability of the models in forecasting disease behavior over specific periods. It is crucial for effective public health planning.^[32]^

### 3.34. Host-to-host transmission models

Host-to-host transmission models concentrate on variables like the rate of transmission, rate of recovery, temporal window of immunological protection against secondary infection and antibody dependent enhancement effect (impact of secondary infection on the entire force of infection) about dengue fever. Temporal Window of Immune Protection is the term used to describe the length of immune protection against any serotype of secondary infection. It has a big impact on how the dynamics of DENV epidemiology are shaped.

In basic host-to-host models, seasonal forcing is used to simulate the impact of vector dynamics. This takes into account how environmental factors affect the spread of disease. Five distinct extensions of the traditional SIR models were used to simulate the dynamics of multi-serotype dengue infections between hosts. This method only considers the actual parameters of a SIR-type model, thereby focusing on the multi-strain component of the disease and its consequences on the host population, while accounting for the effects of vector dynamics.

### 3.35. Finite difference method

This method use finite difference techniques to accurately represent the unpredictable parts of disease transmission and medical consequences, ensuring numerical solutions that are both stable and accurate.^[33]^ It separates the differential equations in time and space, allowing for the modeling of disease dynamics on a grid. The model considers the dynamics of sickness progression in the context of therapy, cure, and partial immunity, as well as stochastic aspects to imitate real-world variability. It guarantees that the numerical solutions remain stable and precise throughout time, which is critical for making dependable forecasts.

### 3.36. Stochastic reaction-diffusion nonlinear chemical model

This methodology improves on existing methods by creating a dependable computational framework that maintains positive and realistic results while increasing stability and consistency.^[34]^ It is frequently used to simulate the spatial distribution of infectious illnesses and chemical responses in biological systems. It improves on existing methods by guaranteeing that solutions are positive and physically acceptable. It shows significant improvements over the classic Euler-Maruyama approach, particularly in regards to stability and consistency. It describes how substances (as well as diseases) disperse through space and respond over time while including randomness.

### 3.37. Stochastic susceptible-infectious-recovered-susceptible model with partial immunity and incidence rate

A traditional epidemiological model was expanded to take into account for partial immunity and varying incidence rates. This robust computational method incorporates stochastic processes into the susceptible-infectious-recovered-susceptible structure, accounting for partial immunity and changing incidence rates, resulting in accurate and stable disease dynamics simulations.^[35]^ It incorporates randomization into the model to represent the unpredictable features of disease transmission. It guarantees that the numerical methods can cope with the complexities brought by stochastic factors while remaining accurate and stable.

### 3.38. Numerical method for stochastic coronavirus model

This model addressing the COVID-19 pandemic, this successful numerical technique uses stochastic aspects to precisely predict the disease’s evolution, assisting in the assessment of intervention strategies.^[36]^ It employs powerful numerical methods to assure accurate and efficient simulations. It assesses the effectiveness of various public health measures, which helps to inform policy choices.

### 3.39. Stochastic dengue model with feature preservation

This approach preserves important aspects of dengue transmission while including stochastic factors, which improves the model’s forecast accuracy and dependability.^[37]^ It describes the fundamental randomness of dengue transmission. It ensures that crucial dengue dynamics properties remain unchanged in the model, even when stochastic aspects are included. It tries to enhance forecast accuracy, which is critical for successful public health planning and response.

## 4. Implications for Covid-19 vaccinations

The study conducted by Kiszewski (2020) expands the analysis to include NIH funding associated with epidemic diseases (dengue, coronavirus, Zika, and Ebola) by employing epidemic threat funding analysis. Research-initiated versus government-initiated patterns are examined and several funding channels^[15,38,39]^ are distinguished in Table 5. Chaudhry (2023) and Manikandan and Govindan (2023) found that include age structure and geographical distribution in models can have a major impact on understanding and managing COVID-19 spread. Differences in vaccination rollout techniques, public health policy, and socioeconomic considerations all contributes to the observed discrepancies.

**Table 5:** Comparison of patterns of investigator-initiated versus government-initiated research for Nth funding related to epidemics.

Aspect	Investigator-initiated research	Government-initiated research
Funding mechanisms	Varied grants and projects	Cooperative agreements, intramural research, program projects, and centers
Pattern of funding	Less consistent	Sustained and coordinated funding
Focus areas	Primarily on specific research projects	Broader scope, including research capacity, program projects, and centers
Contributions to PMIDs and project years	Large fractions of investigator-initiated projects	Majority of funding associated with government-initiated research
Consistency across epidemic threats	Patterns may vary for each disease	Similar patterns observed across coronavirus, Zika, Ebola, and dengue funding
Success in vaccine development	Relationship between funding amount and success not demonstrated	Limited correlation between NIH funding amount and vaccine development success
Observations	A less robust mechanism for vaccine development	Sustained support for various aspects of vaccine development beyond specific projects

NIH = National Institute of Health, PMIDs = PubMed Identifier.

**Figure 7.**
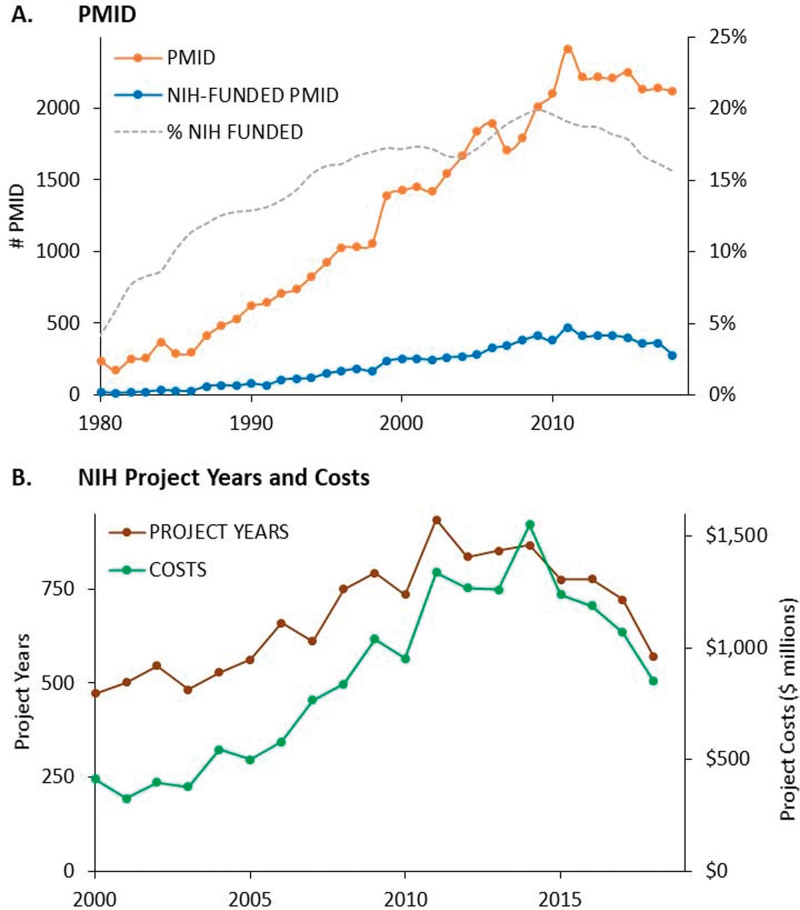
NIH support for published research on vaccine technologies.^[12]^ NIH = National Institute of Health.

It highlights the significance of ongoing NIH funding for pandemic response. A comparison with past difficulties sheds light on the current state of vaccine research. The traditional obstacles of vaccine development, such as cost, safety, and logistical problems, are major barriers that must be overcome to enable effective vaccination campaigns (Fig. 7). Given the tight resources for public health, the fact that many regions rely on nongovernmental organizations like United Nations Children’s Fund or Pan American Health Organization to buy vaccines, and the requirement of mass vaccinations to provide community protection, the demand for public health vaccines has become extremely sensitive to cost.^[15]^

### 4.1. Comprehensive parameter analysis

The extensive parameter analysis and recommended values in Table 6 provide practical direction for model implementation, which is frequently lacking in prior publications. This table makes it easier to replicate and apply our models to a variety of epidemiological scenarios.

**Table 6:** Parameters and variables used in the models.

Variable/parameter	Description	Values
S(t)	Susceptible humans at time *t*	Initial population size 0≤S(t)≤N
I(t)	Infected humans at time *t*	Varies based on initial outbreak data 0 ≤I(t)≤N
T(t)	Treated humans at time *t*	Initially 0
R(t)	Recovered humans at time *t*	Initially 0 0 ≤R(t)≤N
N(t)	Total population at time *t*	N(t)= S(t)+ I(t)+ T(t)+R(t)
$S_{r}\left(t\right)$	Susceptible rodents at time *t*	Initial rodents population size
$I_{r}\left(t\right)$	Infected rodents at time *t*	Varies based on initial outbreak data
$R_{r}\left(t\right)$	Recovered rodents at time *t*	Initially 0
$N_{r}\left(t\right)$	Total rodents population at time *t*	$N_{r}\left(t\right)=\;S_{r}\left(t\right)+I_{r}\left(t\right)+\;R_{r}\left(t\right)\;$
β	Transmission rate from infected to susceptible individuals	0.1 to 1.0 per contact per day
γ	Recovery rate	0.1 to 0.5 per day
μ	Mortality rate	0.01 to 0.1 per day
δ	Rate of progression from exposed to infected	0.1 to 0.2 per day
σ	Rate at which asymptomatic individuals recover	0.05 to 0.2 per day
A(t)	Asymptomatic infected individuals at time *t*	Varies
E(t)	Exposed individuals at time *t*	Varies
D(t)	Deceased individuals at time *t*	Initially 0
Ψ	Interaction parameter between 2 strains	0.1 to 0.5
TCI	Temporal window of immunologic protection	6 to 12 Months
α	Rate of loss of immunity	0.01 to 0.1 per day
k	Force of infection	1.0 to 2.0
$R_{0}$	Basic reproduction number	1.5 to 3.5

## 5. Results

Davydovych et al (2023) underscore the importance of spatial dimensions in mathematical modeling, drawing particular attention to the spatial variability that was witnessed during the COVID-19 pandemic. Understanding the spatial transmission of epidemics is aided by the investigation of several mathematical models, particularly ones that incorporate spatial dynamics. Initiatives and plans related to public health can then be informed by this. To be more precise, the models created in the paper shed light on the spatial dynamics of epidemic activities. Creating focused public health interventions requires an understanding of how diseases spread among various demographics and geographical areas. Chaudhry (2023) anticipate that adding spatial heterogeneity to the models will increase forecast accuracy and make public health interventions more applicable.^[16]^

Issaka (2023) and Cacciapaglia (2022) highlights how crucial it is to take into account more intricate models^[4]^ incorporating age structure and geographical diffusion in addition to more traditional models like SIR and SEIR.^[13,40]^ These models provide a useful tool for epidemiologists and other researchers by enabling a thorough examination of the transmission of epidemics (Table 7). The HAP-Cu PGE/SARS-CoV-2 antibody system provides high detectability, rapid testing, and inexpensive production costs, according to the study’s conclusion. It highlights the system’s ability to reduce illness spread and save time by imagining a user-friendly at-home SARS-CoV-2 detection tool.^[8]^

**Table 7:** Insights from mathematical and epidemiological models.

Disease	Mathematical models	Epidemiological models	Insights
Ebola	Fractal fractional Caputo operator	Compartmental model with susceptible, infected, and recovered compartments includes treatment and mortality dynamics^[16]^	Provides a more accurate and nuanced description of Ebola dynamics
	Various models exploring transmission dynamics and interventions	Population-based models, spatial models	
COVID-19	Involves differential equations and variables	SEIR model incorporates variations for different populations and interventions^[37]^	Enhancing the overall comprehension of COVID-19 dynamics.
	SIR, SEIR, Agent-Based Models, etc	Global, regional, and local models	
Dengue	Mathematical modeling of transmission dynamics SEIR model commonly	The SEIR model is commonly used, considers vector (mosquito) population and human infection dynamics	Aiding in understanding the complexities of dengue spread
	SIR, SEIR, Compartmental Models	Transmission models, vector dynamics	
Monkeypox	Mathematical representation of transmission dynamics, Atangana–Baleanu fractional derivative	SEIR model or variations may include factors related to zoonotic transmission, vaccination, and population demographics	Captures fractional nature in transmission dynamics, providing a refined Monkeypox description.
	SIR, SEIR, fractional derivative models, symmetry model	Transmission models, host interaction, optimal control strategies	

SEIR = susceptible-exposed-infected-recovered model, SIR = susceptible-infectious-recovered model.

Mergenthaler (2022) used a variety of spatial patterns, with experiments exhibiting varying degrees of complexity and quality. The analysis highlights shortcomings in advice and useful insights and addresses how spatial statistics are presented, visualization strategies, and the application of results to public health policy.^[1]^ The presentation of Hasan et al’s findings relies heavily on numerical simulations. This highlights how the behavior of the model is affected by fractional order derivatives. The results are visualized using graphs that show the dynamics of distinct illness classifications under different fractional orders.^[16]^ It explains how simulations show how various disease classes’ convergent traits develop over time. These simulations offer a visual depiction of the behavior of the model in different fractional orders and situations.

These results demonstrate the potential value of validated techniques in mathematical epidemiology as a substitute for dealing with difficulties in real phenomena modeling. It is important to remember that even though VSPODE^[3]^ makes an effort to manage overestimation at every integration step, more investigation may be necessary to develop validated solvers for particular model properties. As a result, those traits can be used to create more effective solutions that lessen the dependency issue.

The development of paper-based electrochemical biosensors for virus detection is a major advancement in the field of diagnosis. The prompt intervention and containment of viral epidemics depend heavily on this decentralized approach. It is in line with the need for quick and easily available diagnostic tools around the world (Table 8). Scientists are still working to improve these biosensors, experimenting with different materials and methods to increase sensitivity and increase the range of viruses that can be detected. These biosensors have the potential to transform virus detection and lead to more efficient disease management techniques as technology develops.

**Table 8:** Advantages of paper-based electrochemical sensor.

Advantages	Description
Accessibility	• Suitable for point-of-care diagnostics **•** Utilized in resource-limited settings • Enhancing healthcare accessibility to conduct on-site virus detection
Low cost	• Cost-effective manufacturing using paper substrates • Affordable diagnostic solutions compared to traditional methods
Accuracy	• Leveraging electrochemical signals for sensitive and precise virus detection • Integration of nanomaterials enhances the biosensor’s overall accuracy
Portability	• Lightweight and portable design for on-site virus detection. • Enables quick deployment in various environments, supporting rapid response. • Allows for efficient virus capture and detection even at low concentrations

While used a compartmental modeling strategy to mimic disease transmission patterns, other investigations used agent-based or mathematical modeling methods. Each technique has advantages and disadvantages for example compartmental models can be helpful for recording population-level dynamics, whereas agent-based models enable individual-level interactions. However, changes in data sources, modeling preconceptions, and parameter estimation techniques may affect the comparability of results. These findings are consistent with prior research, which has found a higher risk of transmission in heavily populated metropolitan settings.

## 6. Discussion

Ekici et al’s study from 2022 focuses on the system’s analytical sensitivity, the effects of different concentrations, and the effective modification parameters. The results demonstrate the electrode system’s potential for quick and affordable SARS-CoV-2 detection.^[8]^ It highlights the importance of extensive and ongoing NIH funding for pandemic response. The study by Kiszewski et al (2020) highlights the deficiency of financing and research concerning epidemic dangers outside of coronavirus for future consideration. Mergenthaler et al (2022) examined the difficulties of incorporating insights into public health decision-making. It explores the need for more precise recommendations, particularly in research utilizing sophisticated analytical techniques.^[1]^ Gurp (2022) examines recommendations for focused interventions, pointing out the widely accepted general approach in disease programs. Ataide et al (2023) emphasized the benefits of paper-based biosensors, including their portability, affordability, and potential in point-of-care diagnostics.^[41]^ Various approaches to improve detection speed, selectivity, and sensitivity are discussed: wearable technology, antifouling qualities, nanomaterials, and microfluidic systems.^[41]^

Hasan (2023) provides a qualitative assessment of the proposed model, equilibrium points, and stability. Akgül (2023) explores the implications of the findings, drawing comparisons between classical and fractional order scenarios and emphasizing the stability of the fractional order derivatives. According to Gunasekar study from 2023 vaccination is the best defense against viral infections. Because of its challenges include ongoing development and supply based on the virus’s structure. Consequently, the goal of sensitivity analysis is to naturally lower the rate of human-to-human transfer. Therefore, the study by Gunasekar and Manikandan (2023) emphasized that using masks, keeping social distance, maintaining good cleanliness, and other preventive measures are some approaches to slow the transmission of the Monkeypox virus. Furthermore, it indicates that to further impede the pandemic’s spread, more afflicted individuals must receive treatment.

Variations in testing techniques, reporting systems, and access to health care may cause differences in disease burden estimations and transmission rates. Variations in population statistics, healthcare infrastructure, and public health programs may also contribute to disparities in outcomes reported between areas. Disease incidence and prevalence rates vary by location due to variances in population density, movement patterns, and adherence to public health initiatives. Variations in healthcare facilities, testing capacity, and surveillance systems could impact the accuracy and dependability of epidemiological statistics. Comparative analyses can provide insights for the creation of focused public health interventions customized to particular geographic, cultural, and socioeconomic situations. Policymakers can use insights from global epidemiological studies to create evidence-based initiatives to reduce disease transmission, improve healthcare infrastructure, and strengthen community resilience to transmissible disease epidemics.

As control variables to stop the spread, the tactics discussed for a novel Monkeypox model created in this work are modified for the symmetry model. Gunasekar et al (2023) presented the Monkeypox viral model, together with characteristics like quarantine status and public awareness campaigns. Additionally, Manikandan and Govindan (2023) offer a model relying on the Caputo-Fabrizo fractional derivative to explain the Monkeypox virus’s exponential-like transmission behavior. Govindan (2023) is a more comprehensive model than previous ones since it includes both medicated humans and recovered rodents having inherent immunity.

For both humans and rodents, this model takes into account different compartments, such as susceptible, infected, treated, and recovered individuals. The interactions between these species and variables like transmission rates, recovery rates, and control methods are taken into consideration in Ahmad and Eman study from 2023. Lizarraide-Bejarano et al’s work from 2022 offers a method for incorporating uncertainty into ODE-based modeling by using local sensitivity analysis, interval arithmetic, and structural identifiability analysis. Lizarralde-Bejarano and Gulbudak (2022) used a model that replicates the spread of dengue illnesses and has 7 state variables and 9 model parameters as an example to show how well these studies worked together. The SEIAR model represents the dynamics of the epidemic more accurately since it takes into account both symptomatic and asymptomatic cases. This model has been especially valuable in understanding the transmission of SARS-CoV-2. It emphasizes the importance of extensive testing and contact tracing to detect and separate out asymptomatic carriers. When this study compare these models to other global studies, such as those by Davydovych et al (2023) and Issaka (2023), which used geographic and age-structured models, it show an increase in forecast accuracy and the applicability of public health interventions. Differences in findings between research can be attributable to varying parameter settings, population demographics, and interaction coefficients.

The research by Champagne et al (2019) considers how mathematical models might be used as narrative devices to shed light on the dynamics of disease transmission. In related research, Champagne and Paulc (2019) examine how knowledge from dengue modeling might help comprehend how COVID-19 spreads, highlighting similarities in characteristics including numerous variations, asymptomatic cases, and the importance of vaccinations.

## 7. Impact of public health initiatives

Intended public health actions require a thorough understanding of how diseases spread among various populations and geographical areas. Researchers hope that adding spatial heterogeneity to the models will increase forecast accuracy and make public health interventions more applicable by assisting in the identification of hotspots for transmission, spatial modeling.^[13]^ Davydovych (2023) makes it possible to take preventative action in high-risk areas to slow the disease’s development. Spatial modeling^[42]^ provides insights that help public health initiatives be optimized by more effectively allocating resources and customizing interventions to particular geographic locations.^[30]^

These models assist in identifying more vulnerable locations, making it possible to implement targeted interventions like immunization campaigns, budget allocation, and the development of healthcare facilities in certain places. By helping to create early warning systems, these models help authorities anticipate and address outbreaks in particular areas, possibly averting or lessening their effects.^[43]^ Policy planning is aided by the insights obtained from these methodologies, which offer a more nuanced knowledge of the various ways that different regions influence the overall dynamics of an epidemic. This encourages the development of public health strategies appropriate for a given region.

Additionally, it can support focused communication tactics, guaranteeing that populations living in high-risk regions are informed specifically about screenings, preventative measures, and medical services. It aids in determining the effect of travel on the transmission of disease^[44]^ and provides direction for decisions on travel bans and quarantine regulations in certain regions.^[45]^ Models help plan for healthcare surge capacity by taking geographical heterogeneity into account, ensuring that hospitals in high-risk areas are ready for an increase in cases.^[46]^

## 8. Recommendations for future research

To expand on the study’s findings and solve the noted limitations, future research should concentrate on a few areas. To begin, longitudinal studies that use primary data gathering methods and novel epidemiological monitoring tools can provide more precise and thorough perspectives on disease transmission dynamics. Furthermore, the development of new modeling frameworks,^[47]^ such as agent-based models or machine learning techniques, has the potential to improve disease model predictive accuracy and scalability. Future research should aim to improve the comparison and generalizability of epidemiological studies by implementing standardized procedures, data sharing efforts, and collaborative research networks. By facilitating international collaboration and knowledge exchange, researchers can improve the comprehension of infectious disease epidemiology, detect emerging dangers, and develop effective disease preventive and control techniques.

## 9. Conclusion

The paper concludes by highlighting the usefulness of various models in mathematical epidemiology as a workable approach. This study recognized that in order to effectively address the issues, different analytical techniques for certain models are needed. Interval analysis research on uncertainty integration and spatial modeling are beneficial to robust modeling methodologies. Spatial modeling enables the identification of transmission hotspots, the enhancement of public health measures, and the direction of region-specific policies. It supports resource allocation, the development of early warning systems, and the expansion of healthcare. Together, the many discoveries strengthen our knowledge of the dynamics of infectious diseases and increase the efficacy of interventions. Additionally, innovations in diagnostic technologies and ongoing financing for vaccine research are essential for successful pandemic response and management. Future study should investigate and modify these models, including fresh data and technological advances to improve their ability to predict and applicability in real-world circumstances.

## Author contributions

**Conceptualization:** Haewon Byeon.

**Formal analysis:** Siva Nanthini Shanmugam.

**Investigation:** Siva Nanthini Shanmugam.

**Methodology:** Siva Nanthini Shanmugam.

**Project administration:** Haewon Byeon.

**Software:** Siva Nanthini Shanmugam.

**Supervision:** Haewon Byeon.

**Validation:** Haewon Byeon.

**Writing – original draft:** Siva Nanthini Shanmugam.

**Writing – review & editing:** Siva Nanthini Shanmugam, Haewon Byeon.
